# Engaging patients in primary care design: An evaluation of a novel approach to codesigning care

**DOI:** 10.1111/hex.12909

**Published:** 2019-05-27

**Authors:** Erin Hertel, Allen Cheadle, Juno Matthys, Katie Coleman, Marlaine Gray, Michele Robbins, Janice Tufte, Clarissa Hsu

**Affiliations:** ^1^ Center for Community Health & Evaluation Kaiser Permanente Washington Health Research Institute (formerly known as Group Health Research Institute) Seattle Washington; ^2^ MacColl Center for Healthcare Innovation Kaiser Permanente Washington Health Research Institute (formerly known as Group Health Research Institute) Seattle Washington; ^3^ Kaiser Permanente Washington Health Research Institute (formerly known as Group Health Research Institute) Seattle Washington

**Keywords:** organizational design, patient‐focused care, primary care, qualitative research, quality improvement

## Abstract

**Objective:**

Recognition is growing that to create truly patient‐centred care, health‐care organizations need to partner with patients around care design. More research into the benefits of engaging patients and the most effective ways of partnering with them is needed.

**Methods:**

This study assessed the process and impact of a collaborative effort to design a new clinic service that balanced the number of patient and clinical provider/staff codesigners involved and recruited patients to represent diverse perspectives. Data sources included interviews with participants, event observation and participant surveys.

**Results:**

Our evaluation found that including patients as equal partners improved the design process by infusing a real‐world, patient perspective. The pre‐event orientation and interactive methods used in the event fostered positive collaboration, as well as personal growth for the patient codesigners.

**Conclusion:**

This study demonstrated the feasibility and benefits of including a roughly equal number of patients and clinical providers/staff in design events and ensuring that the patients represent diverse perspectives.

**Practice implications**

- Our findings and lessons learned can be used to improve partnerships with patients in the design of health‐care services.

## 1 INTRODUCTION

Recognition is growing that to create patient‐centred care, health‐care organizations need to more directly engage patients across the spectrum of health‐care design and quality improvement.^1^, ^2^, ^3^ A useful patient engagement framework developed by Carmen et al includes a three by three matrix that maps three levels of patient engagement: consultation, involvement and partnership/shared leadership across three domains: direct care, organizational design and policymaking.^1^ To date, most patient engagement efforts have focused on the direct care domain, including such activities as patient activation and partnering around shared decision making or self‐management support.^4^, ^5^

Patient engagement at the organizational‐design level has been primarily confined to consultation, using mechanisms such as patient‐experience surveys or patient advisory councils.^2^, ^6^ There have been recent examples of health‐care organizations testing more robust approaches of engaging patients in quality improvement or care design^7^; however, patients are still seldom brought in as partners or coleaders.^3^, ^8^ Those cases where patients *are* engaged at the partnership level have encountered a number of challenges, including clinical staff concerns about involving patients, recruitment and training issues, differences in content knowledge, and perceived power between patients and clinical staff.^8^, ^9^ Even when organizations make an effort to bring patients in as full partners, only a few are typically included, often relegating them to token participation.^7^, ^10^ Overall, the studies that exist report variable benefits of engaging patients as partners and describe a need for more evidence‐based models.^7^, ^11^, ^12^, ^13^

This study presents evaluation results from a care design effort that attempted to engage patients as equal partners in designing a new clinic service in three primary care clinics within Kaiser Permanente Washington, a large integrated delivery system in Washington State. Twelve patients contributed as codesigners in creating the new service—a lay staff person to connect patients with community resources. The new role was intended to support broader, ‘whole‐person care’ by providing access to programmes and services outside of medical services provided by the clinic. Patient codesigners participated in a four‐day design event and a ‘check‐and‐adjust’ event 15 months after the service was implemented.

This paper describes the care design process, including how we recruited, prepared and involved patients, and the impact patients had on the design process and its outcomes. We also present lessons learned that may be useful for organizations interested in engaging patients codesign activities. The design effort was a step in a larger process that included implementing the new role in the three clinics and evaluating its impact on patient care and satisfaction.^14^

## 2 METHODS

### 2.1 Programme description

#### 2.1.1 Design event overview

In the initial 4‐day design event, 27 participants (clinical providers and staff, patients, and facilitators) designed a new clinic service to link patients with community resources. ‘Clinical Providers’ included those providing clinical care (eg doctors, nurses, physician assistants); ‘staff’ included administrative personnel and medical assistants who were part of the primary care team. ‘Patients’ were individuals receiving care from one of the two pilot clinics involved in the study, and ‘facilitators’ were individuals from within Kaiser Permanente Washington's improvement promotion or research teams who helped plan and facilitate the event.

Before the event, the design team worked closely with delivery system leaders responsible for primary care clinic operations to develop a charter that outlined expected outcomes for the design event, participant roles, decision‐making structure and boundaries around what changes were possible to make. A key tenet of the charter was that patients were to be empowered to voice their opinions and participate in group decision making. After 15 months, participants returned for a 3‐day check/adjust event with group review and decision making (day 1) and in‐clinic testing of the new clinic service (day 2‐3).

Both design and follow‐up events followed Kaiser Permanente Washington's Lean continuous improvement model.^15^ It is a team‐driven approach where Lean facilitators bring a few simple improvement principles to teams, and the teams come up with the solutions. At Kaiser Permanente Washington, the method is flexible but typically involves quick, focused workshops. The design event mixed large‐group and small‐group activities to engage participants in decision making. Facilitators added activities to the standard Lean process to ensure that the full group understood and felt comfortable with the role of patient codesigners and their expertise and to emphasize the need to define medical jargon. Patient codesigners were encouraged to play key roles in icebreaker activities, small‐group work, role‐playing, and large‐group report outs, including facilitating morning icebreakers, serving as small‐group leaders and presenting at report outs.

#### 2.1.2 Patient codesigner recruitment and orientation

Patient participants, for the initial design event, were selected from patients assigned to a primary care provider at one of the participating clinics. Letters (n = 349) were sent to a stratified random sample of patients with recent experience at a participating clinic, defined as continuous Kaiser Permanente Washington enrolment, plus at least two face‐to‐face visits in the prior year. We stratified by ethnicity, age, sex, insurance coverage and selected chronic conditions, and oversampled patients who were non‐white, male, on Medicaid and had one or more of the selected chronic conditions. The goal was to ensure that patient participants represented diverse perspectives.

A brief phone screening provided interested patients (n = 23) with information and secured commitment. One patient codesigner was referred by clinic leadership. Twelve committed to and attended the initial design event. Eight of the patient codesigners participated in the check/adjust event. See Table 1 for a summary of the recruitment numbers and demographic information about the twelve patient codesigners.

**Table 1:** Patient recruitment counts and characteristics of patient participants in the design event

Recruitment	Number (%)
Number of letters mailed; of those:	349 (100)
Number of expressing interest	23 (7)
Number of participants: initial design event	12 (3)
Number of participants: check and adjust event	8 (2)
Participant characteristics	
Total participants	12 (100)
Gender	
Female	7 (58)
Male	5 (42)
Race ethnicity	
White	7 (58)
Black/African American	2 (16)
Indigenous/Native American	2 (16)
Asian	1 (8)
Age	
<50 years old	5 (42)
50‐70	5 (42)
70+	2 (16)
Chronic disease (hypertension, diabetes or asthma)	
Yes	9 (75)
No	2 (16)
Unknown	1 (8)
Past/current experience in health care	
Yes	3 (25)
No	9 (75)
Past/current experience in community services	
Yes	7 (58)
No	5 (42)

The design event also included 11 clinical provider/staff codesigners; thus, patients constituted 12 of the 23 (52%) active participants. In addition to patient and clinical provider/staff codesigners, there were five facilitators present, including two experienced Lean facilitators from the delivery system, two delivery system leaders, and the leader of the research project. See Table 2 for a summary of the number of participants by role and project phase.

**Table 2:** Participant and interviewee counts for the design and check/adjust events, by group

Stakeholder groups	Present at design event	Interviewed after design event	Present at check/adjust event^a^	Interviewed after check/adjust^b^	Total interviews
Patient codesigners	12	12	8	8	20
Clinical provider/staff codesigners	11	11	5	0	11
Facilitators	5	4	4	2	6
Total participants	27		17		

a Four patients and all providers from one clinic did not attend the check/adjust event due to a change in the pilot clinic. An additional five provider/staff members attended the check/adjust event from the new pilot clinic but were not included in the table.

b Only patients and facilitators were interviewed after the check/adjust event

Patient codesigners were paid up to $2000 to cover the time they spent in the design meetings, trainings and reviewing materials, with the total payment based on the estimated hours of commitment at $20 per hour. Clinical providers and staff participated in lieu of regular clinic duties as is standard for Kaiser Permanente Washington care design.

To promote meaningful patient partnership, significant attention was paid to orienting patient codesigners before the process began. An interactive orientation session was held at each of the two clinics participating in the study 1‐2 weeks before the design event to provide patient codesigners with information about clinic processes and the primary care team. These orientations were 4 hours in length and included: an explanation of their role and expectations for participation, a clinic tour to familiarize patient codesigners with behind‐the‐scenes clinic activities, review of some clinical terms and time to get to know fellow patient codesigners.

### 2.2 Evaluation methods

We used a largely qualitative approach to assess the impact of patient codesigner engagement on the design event process and outcomes. Data sources included interviews with participants, event observation and participant surveys. The study was reviewed and approved by the Kaiser Permanente Washington Health Research Institute Institutional Review Board.

#### 2.2.1 Participant Interviews

The evaluation team (EH and JM) conducted interviews with all the non‐research team participants (n = 27) after the first design event; with the 8 participating patient codesigners; and two of the facilitators after the second event (see Table 2 for interview counts by role). The interviews captured participant perceptions of the patient codesigner role, degree of engagement/collaboration and patient codesigner impact on the process and final design. Interviews were conducted by the evaluation team via telephone or in person and were audio recorded and transcribed. The interview length ranged between 20 and 60 minutes.

#### 2.2.2 Observation and survey data

The evaluation team (EH and JM) observed the design events using a tool developed by the research team to capture information on content, group collaboration, facilitation, and level of patient codesigner engagement and input into the design. Design event participants completed a short, anonymous survey assessing experience, satisfaction, challenges and perceptions at the conclusion of each day.

#### 2.2.3 Analysis

Interview transcripts were coded using a modified template approach.^16^, ^17^ Themes were derived from a priori topics and inductively. An initial code list was drafted based on representative transcript review. This draft code list was reviewed by a qualitative‐analysis team (EH, CH and JM) and revised. Each team member coded three transcripts. Coded transcripts were compared and discussed. Codes were added and revised, and code definitions were clarified based on questions and coding differences. After a second round with four additional transcripts and another team member (MG), a high degree of comprehensiveness and intercoder reliability was achieved for application of the codes. Remaining transcripts were divided among three team members (EH, JM and MG) for coding, using Atlas.ti to document and manage coded data. After coding, data were organized by specific codes and reviewed. The lead author (EH) drafted coding memos with key findings with example quotes for all codes relevant to this report. Coding memos were reviewed and discussed by the research team.

For observational data, the text was reviewed by two members of the analysis team (EH, JM) who generated a list of preliminary themes that were compared to the key themes highlighted in the interview coding memos to identify areas of similarity and difference. Differences and specific examples were discussed with the qualitative research team to reach shared understanding of observation themes.

## 3 RESULTS

The findings presented here focus on four overarching themes that emerged from analysis of qualitative interview and observational data—overall impact of including patient partners in the design process; the collaboration experience; patient expertise and unique contributions; and the impact of the design event on participants.

### 3.1 Impact of patients on the design process

Most respondents stated that involving patients in the design event influenced the design of the new service; as one participant stated, ‘…I think they had their fingerprints on everything’ (Facilitator #24). Nearly all respondents said that having patients actively participating in the process changed discussions and activities so that a more patient‐centred design emerged overall. Many respondents described satisfaction with the final product and the process.

‘I think you get a richer product. … I realize just how limited my view is of patients and their well‐being and what they consider important. So, I think you get a broader perspective in terms of that’. Clinical provider/staff codesigner #22

Agreement was strong that patients sharing their care experiences made role‐playing and discussions more tangible and served as a ‘reality check’ for clinical provider/staff codesigners to hear directly from patients about what is important regarding their care. Patient codesigners elevated awareness of how the new service would address patients in their lives outside the clinic.

‘I think expectations grew a little bit for this position, because of the patient advisors' experiences and things that we were looking for. I think that as employees and doctors they see one side, the patient sees another, and to bring them together for both to see each side really was helpful’. Patient codesigner #3

‘One of the things that would come out is, ‘Why do you have to ask us our name four different times?’ … It just brought forth how kind of cold and robotic some of our processes come across. Again, all for very good and valid reasons… but from that sense, it was eye opening’. Clinical provider/staff codesigner #24

The number of patient codesigners was seen as a positive factor that brought a variety of viewpoints and increased patient comfort in participating. Facilitators commented that including 12 patient codesigners reduced the perception that the experience of one patient equally represents all patients, a challenge they had seen in events with only one or two patients.

‘It felt like patients, because they knew there were so many other patients there, they felt empowered and were very free to share opinions, versus …[the events] where they're the only one or two in the room’. Facilitator #26

Many participants commented that having patient codesigners engaged made the designed service more patient‐centred because patients could describe what they needed in a given care situation. Specific impacts noted included comments about where the service should be physically located in the clinic, and diverse community needs and resources that staff participants may have overlooked.

Finally, patient impact was noted by respondents at the ‘check‐and‐adjust’ session held 15 months after the role was implemented, when patients learned about what was and was not working in clinics. Patient codesigners advocated effectively for the use of ‘warm handoffs’ (ie taking the patients in‐person to meet the community resource specialist) to address issues that had surfaced in the referral process. This element had been part of the initial design but de‐emphasized in implementation.

### 3.2 Experiences of collaboration

All respondents described effective collaboration between patient and clinical provider/staff codesigners, many linking this to the egalitarian and democratic atmosphere they felt was fostered at the event. In particular, some participants reported that despite a clear difference in technical expertise between patient and clinical provider/staff codesigners, they did not experience a power differential. All 12 patient codesigners felt their thoughts and opinions were respected during the design workshop.

‘It is astounding to see a doctor working along with a patient—the degree of separation between the two is tremendous, even in our society, and yet here they were working side by side, giving ideas and exchanging ideas, accepting ideas, and it wasn't a case of them telling us ‘oh, this is a lot better to do it this way than this way’. Patient codesigner #6

Clinical providers/staff codesigners reported having some concerns about how the patient codesigners would interact with clinical provider/staff participants or comport themselves overall, especially if they had negative health‐care experiences. In the end, many reported positive collaboration experiences.

‘The first day I was a little concerned because the patients kind of wanted their voice heard and I wasn't sure how we were going to get through all the processes that needed to happen. But… the patients were very helpful in some decision making…they really wanted to understand how we do things and/or what are some of our barriers so that they could help design the role. …The first day I was like oh my goodness, we're not going to get through any of this work. So yeah, at the end I was quite pleased’. Clinical provider/staff codesigner #20

Participants thought that interactive aspects of the event were important in engaging patients in the process. In particular, participants highlighted role‐playing for focusing on the patient perspective and small groups for engaging a diverse group of people, including patients who may feel more comfortable speaking in a smaller group. Participants recognized the need for good facilitation to meaningfully engage patients in this type of event and ensure that all voices were heard.

‘I think the role‐playing helped me out tremendously. I always find role‐playing to be a good tool because it lets people relax, it lets people loosen up and be more open than just direct talk’. Patient codesigner #5

### 3.3 Patient expertise and experiences

Participants described how patient codesigners brought a different kind of expertise than clinicians, adding value to the design process in two key ways. First, 75% of patient codesigners had experience with significant or chronic conditions and shared their personal care experiences at key moments to bring the patient experience to life. Interjection of patient experience into discussions was seen as critical.

‘I shared a little bit about my feelings and experiences with my doctors directly at [Kaiser Permanente Washington], and how…I was diagnosed with diabetes and that was it …When I [shared], other people spoke up and agreed, and had the same kind of issues…I think that having the information from the patients … it kind of opened their eyes a little bit more’. Patient codesigner #4

‘These [patients] are always here so they really remind you about how you are doing something to make it work well for them… Not like we don't have to think about our own processes and what we can do, but I think just having consumers there really keeps you focused on also what they're experiencing, how all of this is going to influence them as well besides our own workflow’. Clinical provider/staff codesigner #11

Second, an unexpected benefit was patient codesigners with professional or volunteer experience that positively impacted their effectiveness in the design event. Participants appreciated that patient codesigners brought their own expertise to the process, frequently commenting on the level of relevant knowledge about the community and community resources.

‘I heard a couple of people comment like, ‘oh, I forgot that person was a patient’ or ‘I thought they were an employee.’ I thought it was pretty brilliant to create an atmosphere where…you're both drawing on that individualized expertise that only a patient can offer, because they've had that experience, or only a staff person can offer because they've worked in the clinic, but they never became opposing or competitive or divided’. Patient codesigner #2

I think it was pretty good. Because you have a bigger spectrum of ideas and you have people with different skills from outside of health care and they have definitely a lot of skills, a lot of organization skills… so they were very helpful. Clinical provider/staff codesigner #9

The perception of expertise was reinforced during the design event when patients took the same leadership roles as their clinical provider/staff counterparts, leading over half of the opening activities and small‐group report outs.

‘I think their willingness to step up and report out for groups. Even me personally, I feel like I often don't have quite the expertise, that I should let someone else with more expertise do that. … I just was so impressed with the patients' willingness to take on leadership in that way’. Clinical provider/staff #23

### 3.4 Impact of the event on patient codesigners

In addition to developing a more patient‐centred product, some patient codesigners described a sense of personal growth or satisfaction from participation. Although some found that use of clinical terminology and jargon could make it difficult to follow all discussions, they described the benefits of learning new skills, interacting with different communities and better understanding how to access care.

‘It not only made me feel more part of the whole group and the process, but it also helped me with some of my fears as far as speaking in front of others. So, it was really awesome’. Patient codesigner #3

The survey data from participants showed that both patients and clinical providers/staff were satisfied with the process and felt they had contributed in a meaningful way. Eighty‐four percent of participants reported being very satisfied with the final outcome of the initial design event. When asked about specific aspects of the experience, patient codesigners and staff reported always or almost always contributing in a meaningful way to the process (97%), that their comments and views were always respected (91%), and that the team always or almost always worked well together (99%).

## 4 DISCUSSION AND CONCLUSION

This study presented evaluation results from a care design effort that attempted to engage patients as equal partners in designing a new clinic service in three primary care clinics. Interviews with patient and provider/staff codesigners and event facilitators suggested that including a significant number of patients in the codesign process had a positive impact on both the process and the ultimate design of the new service. Participants reported that patients helped create a more patient‐centred and higher quality role and that the collaboration between clinical provider/staff codesigners and patient codesigners allowed a more diverse set of perspectives to be taken into consideration. Patients also brought unique experiences and expertise to the discussion that enriched the design process. Some patients found the design event allowed them to develop new skills and develop a new level of confidence in speaking out.

While engaging patients as partners in quality improvement and care design is not a new concept,^18^ there are relatively few studies that provide clear guidance about best practices and expected outcomes.^19^ Our project added two design features that have not been documented in the literature to date: (a) including a significant number of patients (n = 12), rather than 1 or 2 which has been the more common practice,^20^ and (b) using electronic health record data to identify and recruit a sample that reached outside of existing volunteer and advocate groups. The hope was that having more patient voices would both increase patient comfort and improve the diversity of patient perspectives contributing to the design. The results showed that having the strong patient partner representation improved the design process by infusing a real‐world, patient perspective. Additionally, the design process appeared to foster positive collaboration, benefited from the unique and different expertise and experiences of patients and fostered personal growth for patient codesigners.

The remainder of the discussion highlights a few key lessons learned from our project that may be useful for other health‐care organizations considering a patient‐engaged design process.

### 4.1 Lessons Learned

#### 4.1.1 Recruit patients using a clear sampling strategy

Our recruitment strategy was critical to ensuring that patient codesigners represented a diverse range of perspectives. We found that patient codesigners not only contributed from their perspectives as patients, but also brought a wealth of personal and professional knowledge to the design process. This is consistent with other studies that stressed the importance of matching patient experience and background to the content of the care design process to ensure that patient contributions are valuable.^7^, ^21^, ^22^

#### 4.1.2 Create a shared understanding of the patient role among all participants

A key challenge found in the literature is engaging patients as equals in care design therefore taking time early to create a shared understanding of the patient role and the specific expertise that they bring to the table is a key step in the process.^21^ This project was able to do this through the patient orientation process and careful attention to facilitation techniques.

#### 4.1.3 Involve enough patients to have a critical voice in the conversation

Our study clearly demonstrated the feasibility and value of involving more than a few token patients. Other scholars in this area have suggested that involving more patients could change the atmosphere by addressing power dynamics often present between clinical providers and patients, creating a cohort experience that empowers patients to contribute and enabling group understanding that patients are not a monolithic group and bring differing perspectives.^9^, ^22^ Our findings validated these assertions.

#### 4.1.4 Include activities and facilitation that allow patients to participate fully as collaborators within an egalitarian atmosphere

Our design drew heavily on literature that suggested that including a variety of activities encourages patients to share their expertise.^23^ We incorporated icebreaker activities, small‐group work, role‐playing and large‐group report outs in order to create many different opportunities to codesigners to share their insights and opinions. We also encouraged shared leadership and fostered collaboration by having patient codesigners take on key leadership, report out and facilitation roles.^9^, ^21^, ^24^

#### 4.1.5 Be aware of language barriers for nontechnical experts

Jargon and clinical terminology presented a significant communication challenge despite our attempts to address it in the patient orientation sessions. One possible solution might be to brainstorm a long list of technical terms that might come up in conversation and create a glossary and activities to help patient codesigners become familiar with the new terminology.

## 5 LIMITATIONS

A few limitations should be noted. The work was carried out in a large, integrated US health‐care system, and the results may be different in smaller systems and those outside of the United States. Study resources offset some of the costs of implementing the patient engagement activities, including patient compensation, recruitment and orientation, and those resources would need to be found in a system replicating the process.

## 6 CONCLUSION

This study incorporated several unique elements into a collaborative care design process, including having a large number of patient codesigners and systematically recruiting them to bring diverse perspectives to the process. We hope that our findings and lessons learned can help support the greater and more effective use of partnerships with patients in care design and shape future research aimed at understanding the benefits of patient engagement.

## CONFLICT OF INTEREST

The authors have no interest to declare.

## DATA ACCESSABILITY

Data sharing is not applicable to this article as no new data were created or analysed in this study.
